# Liquid Anaerobic Digestate as Sole Nutrient Source in Soilless Horticulture—Or Spiked With Mineral Nutrients for Improved Plant Growth

**DOI:** 10.3389/fpls.2022.770179

**Published:** 2022-03-23

**Authors:** Kristina Weimers, Karl-Johan Bergstrand, Malin Hultberg, Håkan Asp

**Affiliations:** ^1^The Federation of Swedish Farmers, Horticulture (LRF Trädgård), Höör, Sweden; ^2^Department of Biosystems and Technology, Swedish University of Agricultural Sciences, Lomma, Sweden

**Keywords:** bio-based society, biogas residues, hydroponics, nutrient solution, organic fertilizer, phosphorus, sulfur

## Abstract

Digestate from biogas production high in plant-available macro- and micro-nutrients could replace mineral fertilizer in protected (soilless) horticulture. Previous uses of digestate have shown that low concentrations of plant-available phosphorus (P) and sulfur (S) may be limiting factors for growth when using digestate as the sole fertilizer. In this study, digestate collected from a municipal biogas plant in Sweden was nitrified in a moving-bed biofilm reactor prior to its use as fertilizer. A greenhouse pot trial with pak choi grown in peat-based growing medium was established to assess the (i) macro- and micro-nutrient availability in the digestate, with particular focus on P and S and (ii) the effect of amending the digestate solution with nutrients considered to be lacking [P, S, magnesium (Mg), manganese (Mn), boron (B), and molybdenum (Mo)]. The results showed that plants fertilized with raw digestate suffered from S and B deficiency and early P deficiency. Supplementing the digestate with nutrients originating from mineral salts resulted in sufficient plant tissue concentrations of all elements except S. The marketable yield was similar to that achieved using standard mineral fertilizer and the dry matter yield was 17% higher. In the light of the present results, the use of nitrified digestate in soilless plant production seems like a fruitful way forward to recycle organic nutrients from waste streams. In the case where a strict organic protocol is not needed, amendment with inorganic nutrients may be a way to increase the utilization of organically derived nutrients.

## Introduction

Anaerobic digestion of organic residues in biogas plants produces renewable energy and a residue containing nutrients essential to plant growth which is known as digestate. The digestate can be used as a plant fertilizer directly or after further processing, contributing to the closing of global energy and nutrient cycles (Alburquerque et al., 2012; Möller and Müller, 2012). According to Jain et al. (2019, and references therein), digestate could replace 5–7% of the inorganic macro nutrient fertilizer currently used globally if the potential of the biogas industry were fully utilized. Digestate is generally spread directly onto fields, like manure (Odhner et al., 2015). However, due to the high share of plant-available macro- and micro-nutrients, e.g., the nitrogen (N) content in a digestate from a mixed feedstock may typically be around 0.5% of the dry weight as was the case in the digestate used in this study (see “Materials and Methods” section), digestate also has the potential to replace mineral fertilizers in protected horticulture (soilless systems). This would increase the nutrient composition requirements for digestate because, unlike fertilizers in soil-based systems, fertilizers in soilless systems must provide the crop with all essential macro- and micro-nutrients at sufficient levels during the whole cropping cycle. To date, research on digestate fertilizers in protected horticulture has been scarce and the results are conflicting. The nutrient use efficiency and harvest when digestate was used compared to commercial fertilizers have been reported to be both higher and lower due, e.g., to different origins of the digestates (Guilayn et al., 2019) but also often dependent on the control treatment used in different studies (Liedl et al., 2004a,b; Ronga et al., 2019; Pelayo Lind et al., 2020). Other findings where results conflict are, e.g., the benefit of the nitrification of the digestate (Uchimura et al., 2014; Pokhrel et al., 2019; Cheong et al., 2020), availability of phosphorus (P) in the digestate (Stoknes et al., 2018), and the effects of adding inorganic nutrients to the digestate (Liu et al., 2009, 2011).

During anaerobic digestion, biogas, primarily composed of methane (CH_4_), and carbon dioxide (CO_2_), is produced through bacterial degradation of organic matter. The resulting digestate is a complex matrix of partially degraded organic matter, inorganic compounds, and microbial biomass (Möller, 2015) in proportions depending on the composition of the biomass feedstock and process parameters, e.g., operating temperature and average retention time (Alburquerque et al., 2012; Risberg, 2015). According to a review by Möller and Müller (2012), most plant nutrients in the raw feedstock are retained during the digestion process, and digestate normally contains all essential macro- and micro-nutrients in varying proportions, reflecting those in the feedstock. Due to mineralization and carbon removal during microbial anaerobic digestion, digestate is characterized by high ammonium nitrogen (NH_4_-N) to total nitrogen ratio, alkaline pH (7.3–9.0), and increased solubilization of essential plant nutrients (Möller and Müller, 2012).

The alkaline pH in digestate can decrease the bioavailability of P and calcium (Ca), due to the formation of insoluble, complex Ca-P compounds, mainly hydroxylapatite (HAp, Ca_5_(PO_4_)_2_OH) (Güngör et al., 2007; Marti et al., 2008). The amount of HAp formed in relation to more reactive plant-available compounds, such as struvite (NH_4_MgPO_4_⋅6H_2_O) and simple calcium phosphates, is partly determined by the amount of magnesium (Mg)^2+^ and Ca^2+^ present in the solution (Nelson et al., 2003; Hjorth et al., 2010; Vanden Nest et al., 2021). Multiple studies have shown that low levels of available P are a factor limiting growth when digestate alone is used as fertilizer (Svensson et al., 2004; Liu et al., 2011; Abubaker et al., 2012; Losak et al., 2014; Stoknes et al., 2018; Pokhrel et al., 2019). To deal with low P availability, some researchers have supplemented digestate nutrient solutions with mineral P, resulting in increased yields of lettuce and kohlrabi (Liu et al., 2011; Losak et al., 2014).

Anaerobic digestion has also been shown to decrease the sulfur (S) content of the feedstock through emissions of hydrogen sulfide (H_2_S) and other volatile S-containing compounds (Massé et al., 2007; Peu et al., 2011; Wahid et al., 2018; Fontaine et al., 2020). Hydrogen sulfide has corrosive properties and must be removed from the biogas stream using one of the varieties of techniques available (Moestedt et al., 2013). In large-scale plants, S-containing gases are commonly removed through the addition of iron (Fe) salts to the digester, resulting in the precipitation of dissolved sulfides with ferric or ferrous iron, which limits the formation of H_2_S (Moestedt et al., 2013). Iron sulfides are insoluble in water and strongly decrease the plant availability of S.

In addition to P, S, and Ca, other nutrients in digestate may also be present in insufficient levels or forms not available to plants depending on the composition of the feedstock to the biogas plant and process parameters. For example, low levels of potassium (K) in digestate have been reported, as well as significant losses of the plant available micronutrients manganese (Mn), zinc (Zn), and copper (Cu) during digestion (Bloomfield and McGrath, 1982; Massé et al., 2007; Marcato et al., 2009; Abubaker et al., 2012; Zirkler et al., 2014). The yield of lettuce and tomatoes has been found to increase after the addition of Fe and Mg, respectively, to digestate (Liedl et al., 2004b; Liu et al., 2011).

When digestate is used as fertilizer in systems lacking nitrifying microbiota from the soil, the high NH_4_^+^-N content in the digestate can result in NH_4_^+^ toxicity, with negative impacts on growth and biomass production. The toxicity to NH_4_^+^ is mainly found when soilless- or various hydroponic production systems are used (Liedl et al., 2004a; Neal and Wilkie, 2014; Ronga et al., 2019). Accordingly, the bacterial oxidation of NH_3_ (in equilibrium with NH_4_^+^) to nitrate (NO_3_^–^) in bioreactors prior to application or in integrated biofilters in the system is recommended and is reported to result in yields similar to those produced using commercial fertilizers in e.g., tomato, lettuce, and pak choi (Stoknes et al., 2018; Pelayo Lind et al., 2020).

In the present study, the plant availability of macro- and micro-nutrients in liquid anaerobic digestate was determined. The digestate was collected from a municipal biogas plant and nitrified in a moving bed biofilm reactor (MBBR) prior to use as fertilizer in the production of pak choi grown in limed peat. Plant growth, quality, and physiological parameters were studied and compared with those of pak choi plants produced with standard fertilization using mineral nutrients only. Special attention was given to the plant uptake of P and S. The experiments were designed to compare the growth performance of plants fertilized with an anaerobic digestate to plants in a mineral fertilizer made to resemble the nutrient composition in the digestate. In another comparison, the anaerobic digestate was amended with mineral nutrients: P, S, magnesium (Mg), Mn, boron (B), and molybdenum (Mo), considered low when compared to the concentrations in commercial mineral fertilizer. The questions to be answered by the study were if the minerals, especially P and S, were taken up as effectively by the plants from the organic fertilizer as from the mineral one; or if a less effective uptake of these elements was the case, due to the reasons discussed above. Also, the overall performance of the plants i.e., growth and mineral content was a question of interest since the presence of several organic compounds may interfere with the plant’s physiology and biochemistry as well as on the availability of nutrients. The hypothesis, based on the intention to measure the plant-available nutrients, is that the plants given the organic fertilizer will perform equally well as the ones receiving a mineral fertilizer, despite the different origin of the fertilizers and the different complexity of the matrices in the solutions.

## Materials and Methods

### Plant Material and Growing Conditions

A greenhouse study was conducted between May and June at the Department of Biosystems and Technology, Swedish University of Agricultural Science, Alnarp, Sweden. On April 30, pak choi (*Brassica rapa*, ssp. *chinensis*, “Joy Choi,” Olssons Frö AB, Helsingborg, Sweden) seeds were sown in a plug tray, with one seed per plug in the same growing media as described below. The emerging plantlets were sub-irrigated and fertilized with half-strength commercial inorganic fertilizer for soilless production (0.5 + 0.5 g L^–1^, respectively, of Calcinit™ and Kristalon™ Indigo; Yara, Oslo, Norway). Two weeks after sowing, the plantlets were transferred to 2-L pots with trays. A growing medium of peat moss (0–25 mm, H2-4, H5-7; SW Horto AB, Sweden) with 5.5 kg m^–3^ dolomite lime [CaMg(CO_3_)_2_], (54% liming effect compared to pure CaO, Björka mineral AB, Sweden) giving a pH (H_2_O) of 6.1, was used in all treatments. The dry bulk density was 284 g L^–1^ and the porosity was 72%. To get the same amount of growing medium to each pot, it was weighed to give two L based on the peat bulk density (EN13040:2007). The pots were kept in a greenhouse compartment where the temperature was set to 18°C and the roof ventilation was opened at 20°C. The greenhouse shading screen was closed when the outdoor light intensity was above 1,200 W m^–2^ s^–1^. Only natural light was provided, giving a weekly mean of 150 MJ (PAR) m^–2^ (Priva Intégro v. 730 + Priva Office, Priva, De Lier, the Netherlands). The plants were irrigated with tap water according to need, which was every seventh day at the beginning of the experiment and once a day by the end of the experiment. Water was manually slowly added into each pot until the drained water covered the trays with 5 mm water. The water was later sought up by the plants and no water was lost through drainage. The plants were harvested on 19 June, 51 days after sowing.

### Fertilization Strategy

The N requirement of *B. rapa* “Joi Choi” was calculated using an estimated shoot fresh matter (FM) yield of 250 g per plant, 30% weight addition for root FM, 95% water content, and 3.5% N content in dry matter (DM), resulting in an estimated N assimilation of 570 mg plant^–1^ for the whole cultivation time. It was assumed that 15% of the added N remained unavailable to the plants (30% of the original digestate consisted of non-mineralized N, as can be seen by the Kjeldahl analysis, an assumption was made that half of this could be mineralized during the nitrification process and later in the growing medium), resulting in an estimated N requirement of 650 mg plant^–1^. Thus, the common basis for the four different fertilizer solutions used in the experiment was the total N addition to the treatments, with all receiving a total of 650 mg N plant^–1^. The plant-available N content in the peat was 20 mg L^–1^. Beginning 3 days after planting, the plants were fed a nutrient solution every second or third day for a total of 13 occasions. The nutrient dose was increased stepwise during the cultivation period, with a starting dose that was half the final dose. The nutrient solutions were stored at 5°C before being used as fertilizers.

### Mineral Fertilizer

A slightly modified version of Sonneveld and Straver’s (1994) nutrient solution, formulated to optimize the growth of Asiatic vegetables including pak choi in hydroponic systems, was used as a reference (M2 in Table 1; Sonneveld and Straver, 1994; Bergstrand and Hultin, 2014). The relative proportions of the nutrients by weight, with the total N set to one, were: NO_3_-N 0.93, NH_4_-N 0.07, P 0.2, K 1.37, Ca 0.64, Mg 0.15, S 0.18, Fe 0.012, Mn 0.003, Zn 0.0017, B 0.00014, Cu 0.0002, and Mo 0.00024 (Sonneveld and Straver, 1994; Bergstrand and Hultin, 2014). The solution was diluted to give a final concentration of 250 mg N L^–1^ to match the concentrations in the digestate treatment. The final amounts added to the treatments are shown in Table 2.

**TABLE 1:** Fertilizer treatments in the soilless production of pak choi and the main variables tested.

Treatment			Variable tested
*Digestate treatments*	D1	Nitrified digestate.	
	D2	Nitrified digestate + P, Mg, S, Mn, B, and Mo, to resemble the nutrient composition of M2.	Compared to D1: The effect of added mineral nutrients on plant growth.
*Mineral treatments*	M1	Mineral nutrient solution designed to mimic the total nutrient composition of D1.	Compared to D1: The plant availability of nutrients in the nitrified digestate.
	M2	Standard mineral nutrient solution, designed for optimal growth.	Compared to D2: The plant availability of added mineral nutrients in the nitrified digestate.
*Negative control*	W	Water	Negative control.

**TABLE 2:** Total amounts of nutrients (mg) supplied to each plant during the cultivation time, as nitrified digestate (D1 and D2) or mineral fertilizer (M1 and M2), in the different treatments (in total, 2.6 l of respective nutrient solution per plant).

	Treatment				Growing medium
		
	Plain digestate	Amended digestate	Mineral digestate equivalent	Standard mineral solution	CaMg(CO_3_)_2_ (liming)
	D1	D2	M1	M2	
NH_4_-N	230	230	214	43	
NO_3_-N	420	420	432	605	
Tot N_min_	650	650	646	648	
K	1,241	1,241	1,243	885	
P	97	**128**	101	128	
Ca	144	**170**	36	413	2,391
Mg	10	**41**	10	100	1,450
S	54	**115**	54	116	
Cl	327	327	331	0	
Na	145	145	24	101	
Fe	59.1	59.1	59.1	8.05	
Mn	0.95	**2.26**	0.96	2.26	
Zn	1.68	1.68	1.68	1.08	
B	0.11	**0.89**	0.11	0.89	
Cu	0.59	0.59	0.59	0.13	
Mo	0.02	**0.16**	0.02	0.16	
Ni	0.02	0.02	0	0	
pH	7.7	7.6	7.6	5.9	6.1

*The pH of the nutrient solutions and growing medium (pH-H_2_O). The total amount of Ca and Mg in the growing medium of each pot, provided by the dolomite lime. The numbers highlighted in bold show which minerals were increased by addition to the digestate in D2.*

### Anaerobic Digestate

Biogas digestate was collected at the Karpalund municipal biogas plant in southern Sweden in February. The feedstock entering the biogas plant consisted of 37% organic household waste, 29% manure (2/3 pig manure and 1/3 cattle manure), 21% slaughter waste, 5% fat from grease separators, 8% other food waste, and < 0.3% iron chloride and iron sludge as processing aids. The average temperature during digestion was 44°C; and the retention time in the reactor was 50 days.

After sieving through a 0.8 mm mesh, the nutrient content in the digestate was analyzed by an accredited laboratory (Eurofins Environment Testing, Sweden AB, Lidköping) using the Kjeldahl and Devarda methods for the total-N, the Kjeldahl method for NH_4_-N (Standard Methods 4500-N mod.) (APHA, 1998), silver nitrate titration for Cl, and, for the remaining substances, extraction with aqua regia (HNO_3_ + 3 HCl) and determination of concentrations by inductively coupled plasma atomic emission spectroscopy (ICP-AES), in accordance with International Organisation for Standardisation (ISO) 11466. The nutrient content in the digestate per kg^–1^ (fresh weight) was as follows: in g kg^–1^ total-N 5.3, NH_4_-N 3.7, P.25, K 1.5, Ca 0.7, Mg 0.045, S 0.28, Na 0.8, and Cl 1.8 and in mg kg^–1^: Fe 325, Zn 9.25, Mn 5.25, Cu 3.25, B.6, Mo 0.1225, and Co 0.05. The total solids content was 2.5% and the pH was 8.1.

The digestate was sieved through a 0.8 mm mesh and nitrified in an aerated small-scale MBBR prior to the experiment, in order to lower the NH_4_-N/NO_3_-N ratio, as described by Bergstrand et al. (2020). A 120 L plastic container served as a reactor. It was filled with 70 L distilled water and 18 L biofilm carriers (K3, AnoxKaldnes, Lund, Sweden) taken from an ongoing nitrification with the same digestate as used in this experiment. The digestate was loaded automatically into the reactor in addition, portioned to keep the NH_4_^+^-N concentration below 2 mg L^–1^. The pH was kept at 5.6–5.8 by the addition of the raw digestate or K_2_CO_3_ when the pH fell below 5.6. A Hach DR1900 spectrophotometer was used to monitor the concentrations of NH_4_^+^, nitrite (NO_2_^–^) and NO_3_^–^ during the nitrification process (Hach Lange tests LCK 303 for [NH_4_^+^], LCK 342 for [NO_2_^–^], and LCK 340 for [NO_3_^–^]) (Hach, Loveland, CO, United States). The maximum conversion of NH_4_^+^ to NO_3_^–^ (11.7 g N m^–3^ d^–1^) was found on day 28 of the process. The nitrification process lasted for 51 days. At the end of the process, water and concentrated un-nitrified digestate was added to reach a final N_min_ concentration of 250 mg N_min_ L^–1^, resulting in 35% NH_4_^+^-N and 65% NO_3_^–^ -N. The occurrence of NO_2_^–^ was checked throughout the nitrification process and in the final solution, it was below the detection limit,0.6 mg L^–1^. The final fertigation solution was odorless, clear in appearance, and light brown in color.

### Experimental Set-Up

The experiment was set up as a completely randomized design consisting of five treatments (including the negative control) with eight replicate pots per treatment with one plant per pot. The pots were randomly placed on a greenhouse table and re-randomized twice during the experiment. Four treatments were compared: nitrified digestate (D1); nitrified digestate with the addition of minerals to resemble the nutrient levels in the standard mineral nutrient solution used in the experiment (D2); a mineral nutrient solution designed to mimic the nutrient levels in the nitrified digestate (M1); standard mineral nutrient solution (M2). In addition, a negative control without fertilization was included (W). The treatments and variables tested are listed in Table 1. The treatments were formulated based on their mineral N (N_min_, NO_3_-N + NH_4_-N) content, and all pots (except the negative control) received the same amount of N_min_ (650 mg plant^–1^). Minerals were added to the digestate treatment D2 in the form of the mineral salts MgSO_4_ × 7 H_2_O, CaSO_4_ × 2 H_2_O, H_3_PO_4_, MnSO_4_ × H_2_O, H_3_BO_3_, and Na_2_MoO_4_ × 2 H_2_O. The final concentration was set to equal the concentration in treatment M2. The total amount of nutrients added to the treatments, analyzed by the accredited laboratory (Eurofins, Kristianstad, Sweden) with the standard methods as described above, is shown in Table 2.

The mineral nutrient solutions (M1 and M2) were mixed separately for each treatment and diluted to 250 mg N L^–1^. Sodium hydroxide (NaOH) was used to adjust the pH in M2.

### Analysis

The following data were collected on growing days 45 and 46 using the youngest mature leaf of each plant: (1) Chlorophyll content, using an MC-100 Chlorophyll Meter from Apogee Instruments, Logan, UT, United States and (2) chlorophyll fluorescence, measured with a Pocket PEA Chlorophyll Fluorimeter from Hansatech Instruments, Norfolk, United Kingdom. The chlorophyll content was measured using the method described by Parry et al. (2014). The chlorophyll fluorescence, expressed as the maximum yield of photosystem II (Fv/Fm), was measured after 20 min of dark adaption. The maximum yield of photosystem II (Fv/Fm) was calculated as (Fm – F0)/Fm, where Fm is the maximum fluorescence and F0 is the minimum fluorescence (Maxwell and Johnson, 2000).

The following data were collected after the harvest of each plant: (1) fresh and dry weight (after 3 days at 60°C) of shoots, (2) number of leaves > 10 mm in length, (3) total area of leaves > 10 mm in length, measured with an LI-3100 Area Meter from Li-Cor, Lincoln NE, United States, (4) concentrations of minerals in the shoot plant sap, (5) total mineral content in the shoot DM, and (6) nutrient content, electrical conductivity (EC), and pH in the used growing medium. Measurements 4–6 were performed by an accredited laboratory (LMI AB, Helsingborg, Sweden), using inductively coupled plasma- optical emission spectrometry (ICP-OES) to determine the concentrations of elements. The leaves for the sap samples were taken in the morning from fully turgid plants. The samples were kept in closed plastic bags in the dark until they were analyzed. The plant sap was sampled by pressing the sap out of 100 g of the oldest but fully vital leaves and analyzed after filtration. The nutrient content of the growing medium was measured in Spurway extract, a weak acetic acid (0.018 mol L^–1^) solution (Spurway and Lawton, 1949).

The uptake efficiency of fertilizer P and S (PUE and SUE) was calculated using the partial balance method, i.e., as the ratio of nutrients removed by crop harvest (nutrient content in shoots) to fertilizer nutrients supplied (Fixen et al., 2015). The uptake efficiency of digestate fertilizer P and S as compared to the readily available orthophosphate in the mineral solutions was determined as the ratio of the nutrients taken up from the nitrified digestates to the rate of nutrients taken up from the respective mineral control (D1/M1, D2/M2 denoted PUE_D/M_ and SUE_D/M_).

### Statistical Analysis

One-way ANOVA and Tukey’s honestly significant difference (HSD) test for differences of means, with the CI set to 95%, were used for the statistical analysis of the data from the experiment. Normality was tested by the Anderson-Darling test for normality and homoscedasticity was tested by Levene’s test. Transformation to the square root of the response factor was needed for one set of results, shoot FW in Table 3. The software used was Minitab Express version 15.1.

**TABLE 3:** Growth and quality parameters at harvest of the pak choi grown in a soilless system fertilized with anaerobic digestate (D1, D2) or mineral nutrient solution (M1, M2).

Treatment	Shoot fresh weight	Shoot dry weight	Chlorophyll content	Leaf number	Leaf area	Chlorophyll fluorescence	Water content
	(g) (*n* = 8)	(g) (*n* = 4)	(CCI) (*n* = 8)	(*n* = 4)	(dm^2^) (*n* = 4)	(Fv/Fm) (*n* = 8)	(%) (*n* = 4)
D1	368 ± 16 b	22.4 ± 0.8 ab	29.8 ± 4.0 a	19.5 ± 1.7 ab	30.6 ± 1.9 a	0.81 ± 0.01 a	93.7 ± 0.2 a
D2	402 ± 22 a	24.4 ± 0.9 a	24.2 ± 2.0 b	17.8 ± 0.4 ab	30.5 ± 1.3 a	0.81 ± 0.01 a	94.1 ± 0.2 a
M1	385 ± 11 ab	23.1 ± 2.1 ab	31.0 ± 4.1 a	19.6 ± 0.6 a	29.6 ± 1.6 a	0.80 ± 0.01 a	94.0 ± 0.5 a
M2	393 ± 21 a	20.9 ± 0.5 b	22.3 ± 2.2 b	17.5 ± 0.6 b	28.3 ± 1.9 a	0.81 ± 0.01 a	94.4 ± 0.2 a
W	10 ± 3 c	1.3 ± 0.5 c	24.1 ± 4.6 b	6.3 ± 1.0 c	1.8 ± 0.2 b	0.72 ± 0.06 b	89.4 ± 1.9 b

*Means, given with SD, within each column that do not share a letter are statistically different (P < 0.05).*

## Results

### Plant Growth: Visual Observations

**FIGURE 1:**
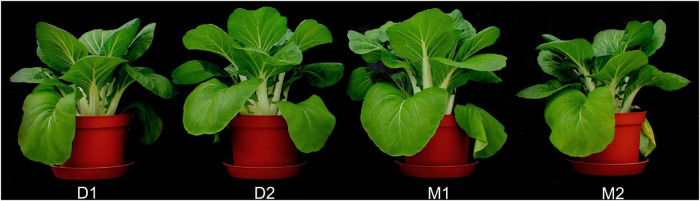
One representative plant from each treatment at harvest. D1, digestate; D2, digestate with amendments; M1, mineral nutrient solution designed to have the same nutrient composition as D1; M2, mineral nutrient solution designed for optimal growth.

At harvest, the plants in all fertilized treatments had reached a height of about 30 cm and showed no signs of nutrient deficiency (Figure 1). There were no obvious visible differences between the plants in the different treatments. The plants in the unfertilized negative control, only irrigated with tap water, had grown to about 10 cm in height at harvest and showed severe symptoms of nutrient deficiency.

### Plant Yield and Physiological Parameters

The plain digestate (D1) and its mineral equivalent (M1) resulted in the same DM yield, the same FM yield, and the same chlorophyll content (Table 3). However, D1 resulted in lower FM yield than the standard mineral nutrient solution (M2). The addition of P, S, Mg, Ca, Mn, B, and Mo to the digestate, giving the D2 treatment, was found to increase the FM yield by 10%, but it did not increase the DM yield significantly. It was also found to decrease the chlorophyll content. The supplemented digestate (D2) performed as well as M2 with respect to FM yield and outperformed it with respect to DM yield (17% higher).

The chlorophyll fluorescence (calculated as Fv/Fm) did not differ between the fertilizer treatments at harvest (Table 3). There were also no differences in the total leaf area between the treatments or in the number of leaves between digestate and mineral treatments (Table 3).

### Nutrient Uptake

The concentration of P in the shoots in D1 was significantly lower than that in its mineral equivalent, M1. In D1, 65% of the applied P was found in the shoots at harvest, compared with 83% in M1, which corresponded to the P uptake from the digestate/uptake from the mineral fertilizer (PUE_D/M_) of 75% (Table 4). The addition of P to the digestate solution significantly increased the shoot P concentration from 2.8 g (D1) to 3.9 g kg^–1^ (D2) (Table 5). This resulted in P recovery similar to that in M2 and a PUE_D/M_ value of 93% (Table 4). The nutrient solutions with the highest P content (D2 and M2) resulted in the highest *P*-values for the shoots and plant sap (Tables 5, 6). The P content in the nutrient solution did not reflect the P content in the growing medium at harvest (Table 7). The M2 treatment resulted in significantly lower residual plant-available P levels in the growing medium at harvest than D2 (1.8 mg L^–1^ compared with 3.3 mg L^–1^).

**TABLE 4:** Nutrient uptake efficiency of phosphorous (P) and sulfur (S) (PUE and SUE), calculated as the ratio of nutrients taken up by crop (content in shoots) to fertilizer nutrients applied (*n* = 4 ± SD).

Treatment	Total amount applied (mg/plant)	Total shoot uptake (mg/plant)	PUE and SUE,%	Concentration in shoots at harvest (g/kg)	PUE_D/M_ and SUE_D/M_
		***Phosphorus***			

D1	97	63 ± 3.1 c	65 ± 3.0 c	2.80 ± 0.75 c	78%
M1	101	84 ± 0.8 b	83 ± 0.8 a	3.65 ± 0.36 b	
D2	128	95 ± 4.8 a	75 ± 3.8 b	3.92 ± 0.27 b	93%
M2	128	102 ± 7.0 a	80 ± 5.4 ab	4.89 ± 0.31 a	

		***Sulfur***			

D1	54	36 ± 3.2 c	67 ± 5.9 c	1.62 ± 0.11 d	71%
M1	54	51 ± 0.6 b	95 ± 1.2 a	2.24 ± 0.25 c	
D2	115	84 ± 2.9 a	73 ± 2.5 bc	3.45 ± 0.17 b	94%
M2	115	89 ± 5.2 a	77 ± 4.4 b	4.26 ± 0.27 a	

*Means within each column that do not share a letter are statistically different (P < 0.05). P (PUE_D/M_) and S (SUE_D/M_) uptake efficiency from the nitrified digestate (D) compared to the mineral solutions (M), were determined as the ratio of the nutrient recovered from the nitrified digestate to the ratio of nutrient recovered from respective mineral control (i.e., D1/M1, D2/M2).*

**TABLE 5:** Concentration of nutrients in shoot dry matter at harvest of pak choi grown in soilless system fertilized with anaerobic digestate (D1, D2) or mineral fertilizers (M1, M2).

	g/kg						
Treatment	N	P	K	S	Ca	Mg	Na
D1	22 ± 6.2 b	2.8 ± 0.1 c	43 ± 1.4 ab	1.6 ± 0.1 d	15 ± 0.9 abc	7.3 ± 0.4 abc	6.6 ± 0.4 b
D2	23 ± 1.6 ab	3.9 ± 0.3 b	37 ± 2.4 bc	3.5 ± 0.2 b	14 ± 0.5 bc	6.7 ± 0.2 c	6.9 ± 0.5 b
M1	27 ± 3.0 a	3.7 ± 0.4 b	47 ± 4.9 a	2.2 ± 0.3 c	14 ± 0.7 c	7.2 ± 0.3 bc	3.9 ± 0.5 c
M2	27 ± 1.8 a	4.9 ± 0.3 a	36 ± 1.6 c	4.3 ± 0.3 a	17 ± 0.8 ab	7.6 ± 0.3 abc	7.0 ± 0.3 a
W	14 ± 2.1 c	0.7 ± 0.3 d	9.4 ± 1.7 d	2.2 ± 0.3 c	16 ± 2 ab	8.1 ± 1.2 ab	9.0 ± 1.5 a

	**mg/kg**						
**Treatment**	**Mn**	**Fe**	**Zn**	**B**	**Cu**	**Mo**	

D1	86 ± 11 bc	49 ± 5.1 a	42 ± 4.7 a	10 ± 0.6 b	4.1 ± 0.4 ab	1.0 ± 0.2 c	
D2	116 ± 1.3 a	52 ± 4.4 a	42 ± 2.1 a	33 ± 1.1 a	4.4 ± 0.6 ab	3.7 ± 0.3 b	
M1	46 ± 3.9 d	53 ± 6.3 a	32 ± 3.3 b	8.6 ± 2.3 b	5.6 ± 1.1 a	1.5 ± 0.2 c	
M2	95 ± 24 abc	55 ± 3.9 a	46 ± 3.5 a	35 ± 3.2 a	3.6 ± 0.7 b	3.4 ± 0.6 b	
W	71 ± 7.8 cd	44 ± 7.6 a	43 ± 2.6 a	8.9 ± 0.7 b	3.4 ± 0.9 b	6.2 ± 0.5 a	

*Means within each column that do not share a letter are statistically different (P < 0.05; n = 4 ± SD).*

**TABLE 6:** Concentrations (mg L^–1^) of macro- and micro-nutrients in plant sap at harvest of pak choi grown in soilless system fertilized with anaerobic digestate (D1, D2) or mineral fertilizers (M1, M2).

	mg L^–1^							

Treatment	NH_4_-N	NO_3_-N	P	K	S	Ca	Mg	Na
D1	6.3 ± 0.7	69 ± 38	86 ± 10 b	2,800 ± 183 a	77 ± 12 b	1,103 ± 159	560 ± 62	290 ± 28 a
D2	5.8 ± 1.0	88 ± 90	185 ± 31 a	2,800 ± 392 a	318 ± 46 a	1,133 ± 158	603 ± 57	338 ± 39 a
M1	6.3 ± 0.8	56 ± 13	120 ± 12 b	2,625 ± 457 a	115 ± 20 b	923 ± 237	523 ± 112	170 ± 22 b
M2	5.4 ± 0.5	37 ± 18	183 ± 25 a	1,725 ± 150 b	283 ± 17 a	1,123 ± 100	613 ± 57	303 ± 55 a
	*n.s.*	*n.s.*				*n.s.*	*n.s.*	

**Treatment**	**Cl**	**Mn**	**Fe**	**Zn**	**B**	**Cu**	**Mo**	

D1	2,200 ± 141 a	6.8 ± 1.4 b	0.93 ± 0.06	3.1 ± 0.32	0.09 ± 0.01 b	0.2 ± 0.03	0.16 ± 0.01 c	
D2	2,150 ± 265 a	10.4 ± 2.2 a	1.17 ± 0.05	3.2 ± 0,33	2.38 ± 0.36 a	0.24 ± 0.03	0.41 ± 0.03 a	
M1	1,750 ± 359 a	3.3 ± 1.0 c	0.96 ± 0.10	1.9 ± 0,17	0.18 ± 0.10 b	0.2 ± 0.02	0.20 ± 0.04 c	
M2	945 ± 247 b	9.1 ± 1.5 ab	1.72 ± 0.96	3.3 ± 1.76	1.88 ± 0.30 a	0.3 ± 0.17	0.32 ± 0.01 b	
			*n.s.*	*n.s.*		*n.s.*		

*Means within each column that do not share a letter are statistically different (P < 0.05; n = 4 ± SD). n.s., no significant differences were found within the columns.*

**TABLE 7:** Plant-available nutrients in mg L**^–^**^1^ growing medium.

	mg L^–1^								

Treatment	N-Kjeldal	NH4-N	P	K	S	Ca	Mg	Na	Cl
D1	2.13 ± 0.52 ab	2.0 ± 0.8ab	2.5 ± 0.6 ab	20.8 ± 5.1ab	3.0 ± 1.4	345 ± 5.8 a	243 ± 5.0 b	51.3 ± 2.3 a	<6
D2	2.45 ± 0.59 a	2.3 ± 0.5a	3.3 ± 0.5 a	22.5 ± 1.7a	4.8 ± 1.0	335 ± 17.3 ab	258 ± 22.2ab	38.3 ± 1.3 b	<6
M1	2.40 ± 0.34 a	2.3 ± 0.5a	1.5 ± 0.6 b	18 ± 2.9ab	3.5 ± 1.0	315 ± 5.8 b	265 ± 17.3ab	26.5 ± 2.7 c	<6
M2	1.16 ± 0.22 b	1.0 ± 0.0b	1.8 ± 0.5 b	15.8 ± 1.3b	4.8 ± 0.5	358 ± 18.9 a	278 ± 9.6 a	26.0 ± 5.0 c	<6
		.			**n.s.**				

**Treatment**	**Mn**	**Fe**	**B**	**pH**	**EC (mS cm^–1^)**				

D1	0.31 ± 0.05 ab	0.45 ± 0.06 b	0.13 ± 0.01 ab	6.7 ± 1.1 a	0.25 ± 0.10				
D2	0.41 ± 0.06 a	0.41 ± 0.18 b	0.13 ± 0.001 a	6.5 ± 0.1 ab	0.25 ± 0.06				
M1	0.21 ± 0.03 b	0.95 ± 0.40 a	0.12 ± 0.001 b	6.3 ± 0.0 b	0.25 ± 0.05				
M2	0.29 ± 0.08 b	0.49 ± 0.06 ab	0.13 ± 0.005 ab	6.6 ± 0.13 a	0.20 ± 0.00				
					n.s.				

*One part of the growing medium was extracted in six parts of 0.018 mol L^–1^ acetic acid for one h and analyzed. pH, and electrical conductivity (EC) in the growing medium at the harvest of pak choi grown in soilless system fertilized with anaerobic digestate (D1, D2) or mineral fertilizers (M1, M2). Means within each column that do not share a letter are statistically different (P < 0.05; n = 4 ± SD). n.s., no significant differences were found within the columns.*

The total uptake of S and shoot-tissue S concentrations were significantly lower in D1 than in M1 (Table 4), with recovery SUE of 65 and 95% in D1 and M1, respectively. The corresponding SUE_D/M_ in D1 was 71%. The addition of CaSO_4_ and MgSO_4_ to the digestate nutrient solution significantly increased the shoot S concentration from 1.62 g kg^–1^ (in D1) to 3.45 g kg^–1^ (in D2) (Table 5). It also resulted in an SUE similar to that in M2 (Table 4). The nutrient solutions with the highest S content (D2 and M2) resulted in the highest plant-tissue and plant-sap S concentrations (Tables 5, 6). The growing media did not differ significantly in S content at harvest (Table 7).

Overall, the shoot concentrations increased for all nutrients that were added to the digestate, comparing D1 and D2 (Table 5). The exceptions were Ca and Mg, for which the concentrations were already high in all growing media due to the liming material content. The shoot concentrations in the amended digestate treatment (D2) came closer to the mineral equivalent (M2), but P and S concentrations were still slightly higher with the mineral fertilizer. The shoot sap concentrations of the nutrients at harvest showed similar results to the total shoot concentration, on comparing both D1–D2 and D2–M2 (Table 6).

## Discussion

### Phosphorus

#### Phosphorus Use Efficiency as Influenced by Magnesium and Calcium

The total P content in the digestate used in the experiments was in the same range as that normally found in mineral nutrient solutions designed for high yielding soilless production (N:P molar ratio of 6.7:1). For comparison, the commonly used Hoagland lettuce solution for hydroponics has an N:P ratio of 7:1 (Smith et al., 1983) and the mineral reference solution in this trial, M2, had an N:P ratio of 5:1. However, a fraction of the P in the digestate was not readily plant-available, as the PUE_D/M_ in D1 was 75% of that in M1, where P was added to the pots as plant-available orthophosphate (HPO_4_^2–^). The Phosphorus Use Efficiency (PUE) value was also lower than that in previous studies on soil, where the PUE close to that of TripleSuper-P has been observed in laboratory incubation trials and pot experiments with digested (but not nitrified as in the present study) animal slurries, energy crops, and mixtures of the two (Loria and Sawyer, 2005; Bachmann et al., 2016, 2011; Vanden Nest et al., 2021). The PUE in the digestate treatment in this trial was also lower than that determined in a hydroponic trial by Pelayo Lind et al. (2020), in which pak choi plants were fertilized with nitrified digestate. In that study, the P shoot concentrations in the digestate treatments were similar to those in the mineral fertilizer reference treatment, but the N:P ratio in the digestate was 3.5:1 and P was probably present in excess amounts.

The reason for the low PUE obtained for the digestate used in the present study might be its low content of Mg and relatively high content of Ca, with a P:Mg, Ca:Mg, and Ca:P molar ratio of about 4, 9, and 2, respectively. The amount of Mg and Ca:Mg ratio is partly decisive for whether more struvite or HAp is formed (Vanden Nest et al., 2021). Despite its low water solubility, struvite is an efficient P fertilizer, resulting in crop PUE comparable to that of water-soluble mineral P fertilizers (e.g., reviewed by Möller et al., 2018 and reported by Vanden Nest et al., 2021). Hydroxylapatite, on the other hand, has low P solubility and plant availability, e.g., a study comparing TripleSuper-P, struvite, and HAp for their P-fertilizing properties in soil found 6.0, 5.4, and 0.7% P recovery, respectively, in the shoots of ryegrass and fescue (Achat et al., 2014). Studies on struvite precipitation in wastewater have revealed that an increased Ca content severely restricts struvite precipitation in favor of HAp formation when the Ca:Mg molar ratio exceeds 1–2.5 (Yan and Shih, 2016; Daneshgar et al., 2018; Liu and Wang, 2019). In manure compost, the formation of HAp is reported to occur when the molar Ca:P ratio exceeds 2 (Toor et al., 2005). For a range of organic fertilizers, including digestate, Vanden Nest et al. (2021) observed a significant negative correlation between P plant availability and molar Ca:P ratio exceeding 2–3, and attributed this to the formation of HAp. Accordingly, it is likely that the low Mg content and relatively high Ca content in the digestate in this study enabled the formation of HAp, with negative impacts on PUE. Two previous studies on the speciation of P precipitates in digestate with relatively low Ca:Mg molar ratio (1.2–1.3) found that, although struvite constituted the major fraction, HAp was present in considerable concentrations. Güngör et al. (2007) found that 78.2% of P in the 25–53 μm size fraction of a dairy manure digestate was present as struvite, and 21.8% as HAp (Ca:P = 2.1), while Marti et al. (2008) found that 58% of precipitated P in anaerobic digestate from a pilot plant was present as struvite and 15% as calcium phosphates, forming mainly HAp (Ca:PO_4_ = 1.6).

#### Phosphorus Use Efficiency as Affected by Fe Supplementation

The addition of Fe-salts for desulfurization in anaerobic digesters can negatively influence plant P availability, as Fe can form insoluble precipitates with P (Krogstad et al., 2005; Möller et al., 2018). Accordingly, increasing the Fe:P ratio has been observed to negatively influence the PUE of organic fertilizers (Vanden Nest et al., 2021). In a trial on P recovery from wastewater, Yan and Shih (2016) concluded that when ferric ions (Fe^3+^) were added at an Fe:Mg molar ratio of 1:5 and in concentrations above 100 ppm, the formation of struvite crystals was negatively affected at pH 7.5 and pH 9.0, probably largely due to the precipitation of ferric phosphates. In the present study, the Fe concentration in the raw digestate was 325 mg Fe L^–1^ digestate and the Fe:Mg molar ratio was 3:1. However, due to the reducing conditions in the biogas process, and an Fe:S molar ratio of about 1:1.5, a large proportion of the Fe added in this study was most probably precipitated as Fe^2+^ with sulfide and present as low-solubility Fe-S compounds (Yekta et al., 2014).

#### Phosphorus Use Efficiency as Affected by Nitrification in a Moving Bed Biofilm Reactor

The precipitation of P as plant-unavailable forms might have continued during storage, due to the slightly alkaline pH (8.1) of the digestate. However, the nitrification treatment in the MBBR decreased the pH to levels where the P solubility is at its maximum (pH 5.5–6.5) for an extended period of time (Hjorth et al., 2010). This potentially allowed for the solubilization of compounds that would otherwise not solubilize within the timeframe of the experiment. Moreover, during MBBR treatment, P speciation is under the influence of microbial processes such as immobilization/mobilization and mineralization. However, the fate of P during nitrification treatments in small-scale reactors or integrated biofilters in hydroponic systems is not well documented and needs further investigation.

#### Risk of Phosphorus Deficiency

The lower uptake of P in treatment D1 resulted in a plant tissue P concentration of 0.28%, which is just within the recommended range (0.3–0.5%) for the optimal growth of *B. oleracea* crops (broccoli, cabbage, cauliflower) (Magnusson et al., 2006). Accordingly, P was not limiting for growth in the digestate treatment. However, the value was just on the verge of potential P shortage, so there may be a risk of P deficiency when using a digestate with a similar or higher N:P ratio than that in this study. The result is in accordance with the findings by Stoknes et al. (2018), who observed the risk of P limitation in tomatoes even after maximizing the P levels by using digestate solids as the growing medium (N:P ratio 1.4:1) and the whole digestate instead of the liquid fraction as the nutrient solution (N:P ratio 6:1). Losak et al. (2014) also observed P limitation of growth when using digestate with an N:P ratio of 6:1 as fertilizer. However, that trial was performed in a soil low in P, where P fixation could be expected (Menezes-Blackburn et al., 2016).

#### Effect of Phosphorus Addition

A straightforward approach to avoid limitations on plant growth and quality when using a digestate with a low content of plant-available P as fertilizer is to supplement the digestate with inorganic P [e.g., phosphoric acid (H_3_PO_4_)]. However, the plant availability of added P is difficult to predict due to the complex matrix in the digestate together with the potentially suboptimal and unstable pH after nitrification due to ongoing nitrification/denitrification processes. Still, active nitrification bacteria will decrease pH when NH_4_^+^ is present but at the same time, anaerobic pockets may form in the growing medium giving suitable circumstances for pH-increasing denitrification (Kremen et al., 2005). The slightly alkaline pH of the digestate nutrient solution in this study (7.6), the relatively high content of Ca (Ca:P ratio 2.1), and the low content of Mg (Ca:Mg ratio 9), posed a risk of precipitation of the added P to poorly soluble compounds such as HAp (Daneshgar et al., 2018; Vanden Nest et al., 2021). However, the recovery of the added P in this study was > 100% (adding 31 mg of extra P per pot resulted in an average increase in shoot P uptake of 32 mg) (Table 3), showing that the (bio)chemical properties of the digestate did not negatively influence the plant availability of the added P. Competing ligands in the digestate, such as the organic ligands citrate and oxalate, which influence the extent to which P ions can bind to metal ions, might have influenced the high recovery (Hinsinger, 2001).

The decrease in digestate pH after application to the pots was probably an important factor for the high P recovery rate. In D2, the growing medium pH was 6.5 at harvest (Table 7). The solubility of P is at its maximum around this pH, as the concentrations of aluminum (Al) and Fe ions on one hand, and Ca ions on the other, are minimized (Lindsay, 1979). There may also have been synergistic effects of P and the other added nutrients. For example, Mo fertilizer is reported to increase P accumulation in the shoots of *B. napus* (Liu et al., 2010). The significantly increased shoot P concentration and P recovery after the addition of P confirm the results obtained by Liedl et al. (2004b), who reported the positive effects of adding H_3_PO_4_ to a pig-slurry digestate. The direct addition of K_2_HPO_4_ into diluted digestate has also been reported to be successful (Liu et al., 2011).

### Sulfur

#### Low Sulfur Content

The total S content relative to N in the digestate was lower than that needed for most crops; the N:S ratio was 12:1, which was twice as high as that in the inorganic control (6:1). In comparison, a hydroponic lettuce solution recipe recommended by various fertilizer companies has an N:S ratio of 7:1 (AkzoNobel, Eurofins Agro, Nmi, Sqm, and Yara, 2016). For *Brassica* crops, which have high S demand and are sensitive to S deficiency (Haneklaus et al., 2007), the optimal ratio is lower. For the *B. oleracea* crops, cabbage, broccoli, and cauliflower, the recommended N:S ratio in the aboveground parts at harvest range between 2:1 and 7.5:1 (Magnusson et al., 2006). If all nutrients are supplied with the fertilizer, a similar ratio of plant-available N and S in the fertilizer is required.

Sulfur (S) and N are both introduced into biogas reactors mainly as a constituent of proteins (Straka et al., 2007). The reactor feedstock in this study consisted of 21% protein-rich slaughterhouse waste and relatively protein-rich pig- and cattle manure and organic household waste. However, due to S losses during anaerobic digestion caused by H_2_S volatilization and/or iron sulfide precipitation, feedstock with a relatively high S content can still result in a digestate with an N:S ratio that is too high to meet the needs of crops in soilless systems (Massé et al., 2007; Peu et al., 2011; Wahid et al., 2018; Fontaine et al., 2020). The S content may also be decreased by volatilization during storage and handling, as digestate can contain potentially volatile S compounds not precipitated with the added Fe (Möller and Müller, 2012).

#### Sulfur Recovery

The use efficiency of S in the digestate fertilizer was 71% of that in the mineral equivalent SUE_D/M_ (Table 4). Considering the reported S-speciation in the digestate, this is a remarkably high value. Yekta et al. (2014) investigated the chemical speciation of S in the digestate from five industrial biogas reactors in southern Sweden which, like the Karpalund reactor, digest mixtures of different organic wastes and use Fe-salts as process additives. They found that the S speciation in the digestate was dominated by insoluble iron sulfides (27–57%), followed by reduced organic S (22–46%) and zero-valent S (6–16%). Inorganic sulfate (SO_4_^2–^), i.e., the form in which plant roots can assimilate S was found in some samples, but only at low concentrations (Yekta et al., 2014). It has been reported that SO_4_^2–^ only makes up 3–8% of the total S in the digestate from anaerobic fermentation of cover crops and straw mixtures (Suzuki, 1999; Fontaine et al., 2020). The amount of iron sulfides entering the Karpalund biogas reactor and the amount leaving the reactor with the digestate used in this study are not known. However, due to the small particle size of iron sulfides, some were probably retained in the digestate.

The mobilization and mineralization of organic S and oxidation of sulfide and elemental S to SO_4_^2–^ are enzyme-driven processes dependent on microbial activity (Edwards, 1998; Suzuki, 1999). The 71% SUE_D/M_ obtained in this study shows that the microflora in the MBBR biofilter, which was transferred to the peat substrate with the digestate, was probably capable of the net mineralization of organic S and oxidizing sulfide and elemental S to SO_4_^2–^. However, it seems unlikely that the oxidation of iron sulfides at any relevant rate took place during the experiment, considering its short duration and the absence of soil microflora (Freney, 1967).

This result is in sharp contrast to the findings in other studies on digestate application to soil, where the S recovery rate has been found to be very low and similar to unfertilized controls, even with the digestate with a relatively high S level and low C:S ratio (Assefa et al., 2013; Fontaine et al., 2020). This has been attributed to high SO_4_^2–^ immobilization after the application of digestate to soil at C:S ratios which are usually related to net S mineralization (<200) (Fontaine et al., 2020).

The high SUE observed in this study supports the findings by Pelayo Lind et al. (2020) of the high uptake of S by pak choi plants grown with nitrified digestate as fertilizer in a hydroponic setup. In that study, the digestate-fed plants outperformed the inorganic control in the uptake of S. Similar to the digestate in this trial, the digestate was from a large-scale biogas plant using iron chloride as a process additive and was nitrified by MBBR. However, their digestate had a lower N:S ratio (6.7:1) than the presently used digestate, probably due to plant-based feedstock with less N. This lower ratio resulted in higher total S application, which explains the higher S uptake compared to that in the present study. However, the reason for the higher uptake may also lie in the hydroponic setup, as the peat growing medium and pot culture in this study allowed for a larger microbial community and thus higher S immobilization, although microbial growth was probably limited by low C availability.

#### Risk of Sulfur Deficiency

The low total S content and the presence of non-plant-available S in the digestate resulted in low plant-tissue concentrations of S in D1 (0.16%) (Table 5). According to Teuber et al. (2020), an adequate S supply is reflected by tissue concentrations of S between 0.17 and 0.40% for most crops. For *Brassica* crops, which are considered particularly sensitive to S deficiency (Haneklaus et al., 2007), the optimum range is higher. For example, Haneklaus et al. (2007) recommended 0.75% for *Brassica* vegetable crops and Magnusson et al. (2006) recommended an S content of 0.4–1.3% in plant tissues for the optimal growth of *B. oleracea* crops. Low levels of S in *Brassica* crops have been found to result in lower yields and lower concentrations of valuable S-containing metabolites such as glucosinolates (Scherer, 2001). Accordingly, the S content in the D1 plants can be considered very low and in the deficiency range, with potential negative effects on yield and quality. The low S availability in the digestate treatments might also explain the lower N concentrations observed in the plants in these treatments, as S interacts closely with N uptake in plants (Eriksen et al., 2001).

Doubling the total S content in the digestate by adding MgSO_4_ and CaSO_4_ to D2 (Table 2) significantly increased shoot-tissue concentration levels of S from 0.16 to 0.36%, which is close to the minimum level recommended by Magnusson et al. (2006) (0.4–1.3%). The recovery of the added S in the shoots was 79% (adding 61 mg of extra S per pot resulted in an average increase in S uptake by shoots of 48 mg). Considering this, a higher S addition rate (e.g., tripling the total S content in the digestate) would have been more beneficial. The sap concentration of S (Table 6), reflecting the vacuolar content of S, was also largely increased by S addition to the digestate, indicating elevated cell S status (Marschner, 1978).

### Effects of Adding Magnesium, Manganese, Boron, and Molybdenum

No correlation was found between the Mg content in nutrient solution and Mg plant uptake, which was probably a result of the high Mg levels in the growing medium due to its dolomite content.

The addition of the micronutrients B, Mn, and Mo to the digestate resulted in significant increases in the shoot mineral content of these micronutrients, e.g., the B concentration increased from 10 to 33 mg kg^–1^ in supplemented plants. For most dicotyledonous species, the critical deficiency range for B is 20–70 mg kg^–1^ (Broadley et al., 2012). For the *B. oleracea* species broccoli and cauliflower, 30–100 mg kg^–1^ B in the shoots has been recommended for optimal growth (Magnusson et al., 2006). Based on these values, the D1 plants suffered from B deficiency and the supplemented D2 plants were just within the range recommended for optimal growth. One of the most rapid responses to B deficiency is the inhibition of root elongation, which results in stubby and bushy roots (Broadley et al., 2012). The low tissue concentrations of B in D1 and M1 (10.1 and 8.6 mg kg^–1^, respectively) may therefore explain the distinctly shorter (but not bushy) roots observed in those treatments (data not shown). However, no aboveground symptoms of B deficiency were detected. Further, there were no differences in DM yield between treatments D1 and D2. This was unexpected, as inhibited shoot growth is a typical early symptom of B deficiency (Broadley et al., 2012). However, the fresh matter yield was significantly higher in D2, which could be explained by an increase in root volume when B was supplied at sufficient levels, allowing for the increased uptake of water. The tissue concentrations of Mn and Mo were above the threshold level for deficiency (10–20 mg kg^–1^ for Mn and 0.1–1.0 mg kg^–1^ for Mo) in both D1 and D2 (Broadley et al., 2012), showing that these nutrients were present in sufficient levels in the digestate.

### General Observations

The sap concentration of all elements added to the digestate (including P and S) increased in D2 compared to D1 (Table 6). In contrast to the total tissue concentration, the sap nutrient concentration is believed to reflect the vacuolar concentration, indicating a larger cellular nutrient buffer when increased, as was the case comparing D1–D2 (Marschner, 1978). The amended digestate in D2 also had the same or higher sap nutrient concentrations compared to the mineral equivalent M2. This could indicate an increased deliverance of nutrients from D2 compared to M2 at the end of the culture time.

The overall good performance by the plants given the digestate, despite the lower uptake efficiency of P and S, may indicate the positive effects of digestates on plant growth beyond the nutrient effect such as the presence of suggested biostimulants such as auxin-like compounds (Scaglia et al., 2017) and humic substances (Guilayn et al., 2020). As a result of the anaerobic digestion of the feedstock, digestates contain a complex mixture of partially degraded organic matter and inorganic compounds, including monosaccharides, free amino acids, fatty acids, polypeptides, nucleic acids, vitamins, phytohormones, as well as compounds of higher molecular weight (Möller and Müller, 2012; Scaglia et al., 2017). The same compounds, when derived from other organic sources, have been reported to act as bio-stimulants on plant growth (du Jardin, 2015).

The chlorophyll fluorescence is often used as a measurement of possible physiological stress affecting plants. All the fertilized treatments had an Fv/Fm at 0.81, showing that they probably were not stressed by any of the treatments or at least all to the same degree. An average value for unstressed vascular plants of various origins has been found to be 0.83 (Demmig and Björkman, 1987).

## Conclusion

As hypothesized, the growth performance in the digestate resembled the growth in the mineral solutions made to mimic the digestate. This was also the case when the digestate was amended with nutrients to resemble a commercial nutrient solution. This suggests that the availability of the nutrients in the digestate is high and that the digestate fully can substitute the mineral fertilizer and with the minor addition of selected nutrients perform even better.

This study made the promising finding that after the addition of macro- and micro-nutrients, nitrified digestate can be used successfully as a fertilizer in the production of leafy vegetables on peat-based growing media. Supplementing the digestate with nutrients increased the FM yield of pak choi by 10% and resulted in similar marketable yields as when using standard mineral fertilizer. Further, plants fertilized with the supplemented digestate had a 17% higher dry weight than plants treated with standard mineral fertilizer. This weight increase was probably a result of P, S, and/or B addition, as shoot-tissue concentrations of these nutrients were low (in the deficiency range for S and B) in plants fertilized with the not amended digestate. However, this study was not replicated over time and thus indicative. More research is needed to identify the factors determining the recovery of P and S in digestate fertilizers. Since struvite is reported to be a readily plant-available P source in organic fertilizers, it would be interesting in future studies ahead to investigate supplementation of digestate with struvite recovered from other waste streams, e.g., sewage sludge or industrial effluents, thus contributing to recirculation of P and closing of global nutrient cycles.

## Data Availability Statement

The raw data supporting the conclusions of this article will be made available by the authors, without undue reservation.

## Author Contributions

KW and HA conceived and designed the experiments. KW performed the experiments and analyzed the data. KW wrote the first draft with HA as the main co-author. KW, HA, K-JB, and MH contributed to the writing and approved the submitted version.

## Conflict of Interest

The authors declare that the research was conducted in the absence of any commercial or financial relationships that could be construed as a potential conflict of interest.

## Publisher’s Note

All claims expressed in this article are solely those of the authors and do not necessarily represent those of their affiliated organizations, or those of the publisher, the editors and the reviewers. Any product that may be evaluated in this article, or claim that may be made by its manufacturer, is not guaranteed or endorsed by the publisher.
